# A Constellation of Atypical Findings in a Nine-Year-Old Child With Dysembryoplastic Neuroepithelial Tumors: A Case Report and Review of the Literature

**DOI:** 10.7759/cureus.27707

**Published:** 2022-08-05

**Authors:** Kyle Tuohy, Jessica Lane, Catherine Abendroth, Mark Iantosca

**Affiliations:** 1 Department of Neurosurgery, Penn State Health Milton S. Hershey Medical Center, Hershey, USA; 2 Department of Pathology, Penn State Health Milton S. Hershey Medical Center, Hershey, USA

**Keywords:** who grade i, case report, glioneural tumor, noonan syndrome, dysembryoplastic neuroepithelial tumor

## Abstract

Dysembryoplastic neuroepithelial tumors (DNETs) are rare, generally benign, mixed neuronal-glial neoplasms occurring most often between 10 and 14 years of age. These lesions are classically cortically based and solitary, found preferentially in the temporal lobe, and most commonly present with seizures. On magnetic resonance imaging (MRI), these lesions are generally cystic and have variable contrast enhancement, which, when present, often involves the periphery. Rarely, lesions followed radiographically may demonstrate delayed contrast enhancement. Here, we present a case of multifocal DNETs involving the cerebellum that demonstrated delayed contrast enhancement. In addition, these occurred in a patient with Noonan syndrome (NS), a “RASopathy” disorder associated with low-grade glial and glioneuronal tumors. We present a summary of all previously reported cases of cerebellar DNETs. Our patient was successfully treated surgically and is doing well clinically, now one year status post his last procedure, and is being closely monitored with serial MRIs for progression. Gross total resection is often curative without adjuvant therapy for most DNETs. Our case emphasizes the importance of radiographic surveillance, as multifocality and recurrence may necessitate more than one procedure. Lastly, clinicians should be suspicious for DNETs and other low-grade glial tumors when treating patients with NS, acknowledging their predisposition for multifocal involvement and atypical presentations.

## Introduction

Dysembryoplastic neuroepithelial tumors (DNETs) are mixed neuronal-glial neoplasms generally considered to be benign (WHO grade I) [1]. The estimated incidence of DNETs is 0.03 person-years per 100,000, peaking between 10 and 14 years and diminishing greatly with increasing age [1]. These lesions are classically cortically based and solitary and are found preferentially in the temporal lobe followed by the frontal lobe. Intraventricular, basal ganglia, and cerebellar locations have also been reported, but are much less common [2].

On magnetic resonance imaging (MRI), these lesions are generally cystic, T1 hypointense T2 hyperintense. They have variable contrast enhancement, which, when present, often involves the periphery. Even more rarely, lesions followed radiographically may demonstrate delayed contrast enhancement [2]. DNETs typically present as intractable epilepsy in children and young adults [3]; however, symptoms may vary based on the location of the lesion. For example, cerebellar DNETs often present with headache, vertigo, ataxia, or gait dysfunction rather than drug-resistant seizures [2].

Herein, we report a unique case of DNETs characterized by multifocality with cerebellar involvement, and with delayed contrast enhancement, in a patient with Noonan syndrome (NS).

## Case presentation

**Figure 1:**
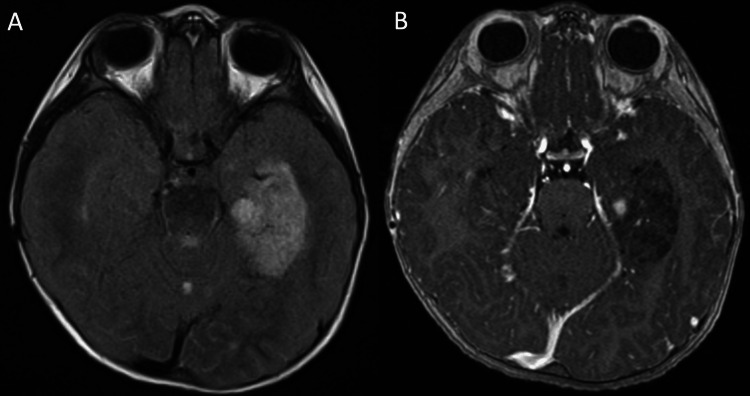
Axial MRI demonstrating left temporal and cerebellar vermis lesions prior to initial resection. (A) T2-FLAIR. (B) Contrast T1.

The patient is a nine-year-old male with NS who presented with seizures. He was diagnosed with intraventricular hemorrhage and Dandy-Walker variant at birth, requiring ventriculoperitoneal shunt placement. He was also found to have congenital heart defects, tethered cord syndrome, and intestinal malrotation. Given these congenital anomalies, he was subsequently evaluated and diagnosed with NS. Genomic evaluation revealed an Asp61Gly variant in the *PTPN11* gene. The patient underwent EEG testing, which demonstrated bitemporal seizure foci. He then underwent MRI imaging, which revealed a flair hyperintense lesion with nodular enhancement in the left medial temporal lobe as well as a small cystic lesion in the cerebellar vermis (Figure 1). He then underwent gross total resection of the temporal lesion and recovered well from this. Pathology revealed a multinodular tumor with focal cortical dysplasia consistent with a DNET.

**Figure 2:**
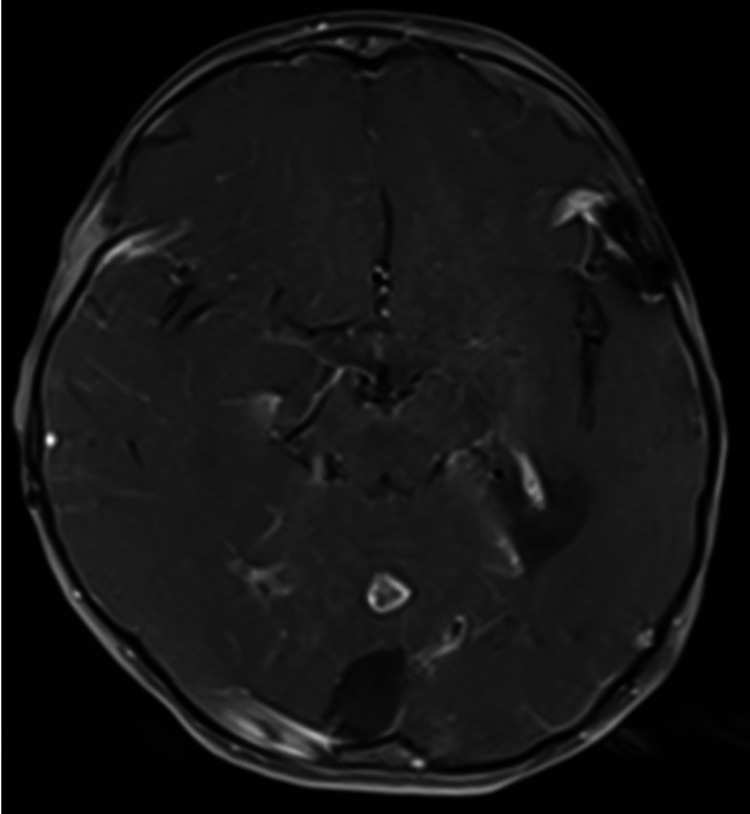
Axial contrast T1 MRI with new enhancement in the previously seen cystic lesion within the cerebellar vermis.

Continued surveillance imaging for the first three years postoperatively demonstrated no recurrence in the temporal tumor bed and mild increase in the size of the non-enhancing lesion in the cerebellum. However, a subsequent MRI performed almost five years after initial resection later revealed ring enhancement in the mass in the cerebellar vermis, which had also continued to grow since prior imaging (Figure 2). Given the significant size increase and new contrast enhancement, surgical excision was recommended for treatment and pathologic diagnosis.

**Figure 3:**
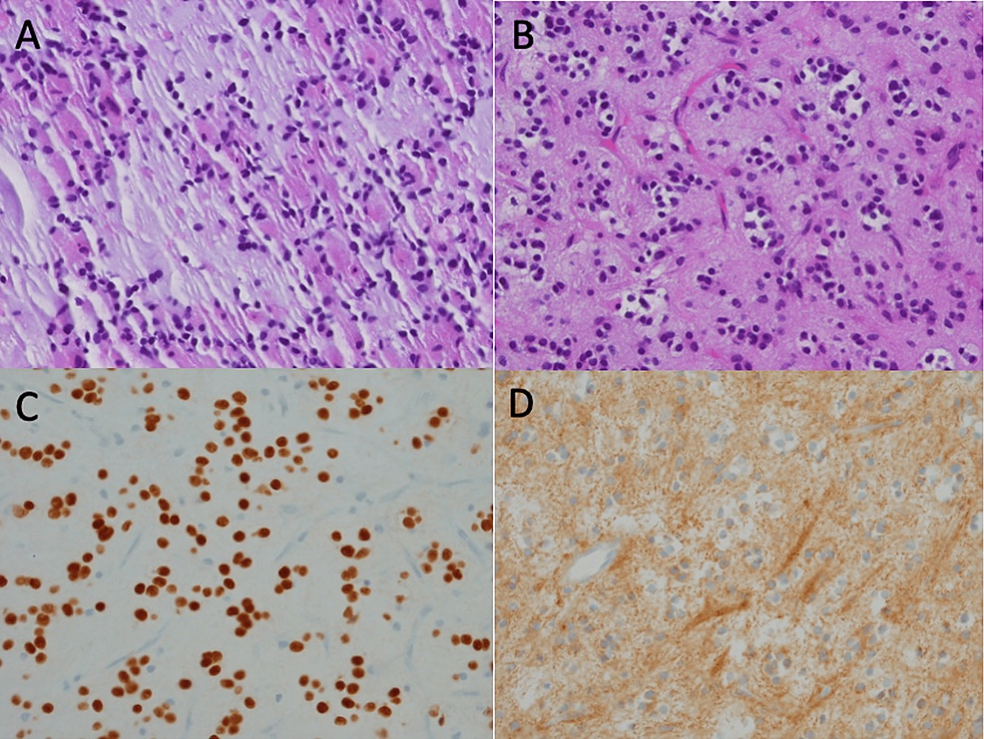
Histopathologic images from cerebellar vermis lesion. (A) Dysembryoplastic neuroepithelial tumor showing nests of oligodendrocyte-like cells and mucinous matrix (H&E frozen section, x500). (B) Dysembryoplastic neuroepithelial tumor showing nests of oligodendrocyte-like cells and delicate vasculature (H&E, x500). (C) OLIG2 immunohistochemistry highlighting nuclei of oligodendrocyte-like cells (x500). (D) NFP immunohistochemistry revealing surrounding neuronal component (x500).

A suboccipital craniectomy was performed using stereotactic navigation and intraoperative ultrasound to confirm the location of the tumor nodule. The area of ring enhancement was entered and found to be relatively avascular, purplish, and friable in consistency. A gross total resection was achieved and confirmed with post-operative MRI, revealing no enhancing area to suggest residual tumor. Pathology revealed oligodendroglial-like cells showing OLIG-2 positivity, embedded in a prominent mucoid matrix, with neuronal elements highlighted by reactivity with GFAP and synaptophysin, consistent with grade I DNETs (Figure 3).

One year later, the patent is doing well clinically. Postoperative MRI revealed no evidence of residual or recurrent enhancement in the cerebellar resection bed. There were some FLAIR hyperintense nodular areas in the left temporal resection bed, which, while appearing stable from the previous year’s scan, were slightly increased in size compared to images four years prior. Given that the pathology from both resections were low grade, and in the absence of any symptoms, we have opted to follow with serial MRIs at this time.

## Discussion

DNETs were first described by Daumas-Duport in 1988 [4], after clinicopathologic analysis of 39 tumors showing features suggestive of dysembryoplastic origin. These tumors typically affect the temporal lobes and present as intractable seizures, accounting for up to 8% of temporal lobe tumors resected for epilepsy [2]. Infratentorial locations, such as the cerebellum, are much less common. A total of 12 cases of cerebellar DNETs have been reported in previous manuscripts, which have been recently summarized [5]. We have identified two additional reported cases of cerebellar DNETs [6,7].

Cerebellar DNETs tend to lack the characteristic imaging signs, and diagnosis must therefore rely on pathological evaluation [5]. Unlike cortical DNETs, which very classically present with seizures, the most common symptoms of cerebellar DNETs include headache, vertigo, ataxia, gait dysfunction, and visual impairment [5]. Interestingly, our patient did not have any clear-cut symptoms relating to his cerebellar lesion, and the decision to resect was made based on radiographic progression.

Contrast enhancement can be seen in roughly one-third of DNETs, and may appear nodular, ring-like, or heterogeneous, and often involve the periphery [2]. More rarely, lesions followed radiographically may demonstrate delayed contrast enhancement, which is thought to result from microvascular proliferation, ischemia, or hemorrhage instead of malignant transformation [8]. Jensen et al. [9] also hypothesized that new enhancement could be a result of increased seizure activity in medial temporal lesions. However, malignant transformation has been described as a rare cause of delayed contrast enhancement [10], often after radiation exposure, although one reported case developed in the absence of adjuvant therapy [11].

Multifocality is also an atypical presentation for DNETs, as these tumors tend to be solitary. However, there have been rare case reports of multifocal DNETs [8,12-17], which was first described by Leung et al. in 1994 [13]. Furthermore, multifocal presentation has been reported in association with hereditary conditions such as Jacob’s syndrome (47,XYY) [14] or neurofibromatosis type 1 (NF1) [18].

Another such condition associated with DNETs, and other low-grade glial and glioneuronal tumors, is NS. This is an autosomal dominant “RASopathy” disorder, affecting genes for proteins involved in the RAS-mitogen-activated protein (MAP) kinase signaling pathway [19]. The pathogenesis of NF1 also involves dysregulation of RAS signaling, which may explain the shared predisposition for malignancies such as DNETs. There are case reports of multifocal DNETs in NS (similar to our case) despite the overall number of case reports of DNETs in NS being small [20]. This may support an association between multifocality and NS.

The most frequent mutation causing NS is in the *PTPN11* gene [20], as seen in our patient. Several variants of *PTPN11* mutations have been described in patients with DNETs [20]. Our patient is only the second case with the Asp61Gly variant in the literature. Interestingly, the other described patient also had multifocal tumors in the temporal lobe and cerebellum [6]. Given the variety of mutations reported in the literature, there does not appear to be a specific amino acid change that predisposes NS patients to DNETs more than another [20]. However, given the low number of patients reported thus far, further research into this association is warranted.

This report helps further characterize DNETs found within the cerebellum, as well as in patients with NS. Given the benign nature of most DNETs, gross total resection is often curative without adjuvant therapy. However, as highlighted in our case, multifocality and recurrence may necessitate more than one procedure. Our case also emphasizes the importance of regular follow-up for these patients to assess for continued growth or new contrast enhancement, given that our patient remained asymptomatic through the follow-up period. Clinicians should be suspicious for DNETs or other low-grade glial tumors when treating patients with NS, with a low threshold for intracranial imaging when a patient presents with the symptoms discussed above. It is also important to acknowledge their predisposition for multifocal involvement and atypical presentations.

## Conclusions

DNETs are a rare form of WHO grade I embryonal neoplasm. Our case is significant for a constellation of atypical DNET findings. First, our patient’s lesion was multifocal, with one focus in the supratentorial compartment and one in the infratentorial compartment. The cerebellar focus was also initially non-enhancing for several years before developing contrast enhancement. Finally, these atypical tumor characteristics were all found in a patient with NS, an autosomal dominant disorder which has been associated with increased incidence of low-grade glial and glioneuronal tumors. This diagnosis should be considered in a child with a cerebellar lesion consisting of cystic and solid components and mixed signal intensities or enhancement. Our case highlights the importance of serial imaging after resection and how the diagnosis of DNETs must still be considered even with atypical imaging findings, especially in patients with congenital RASopathies such as NS.
